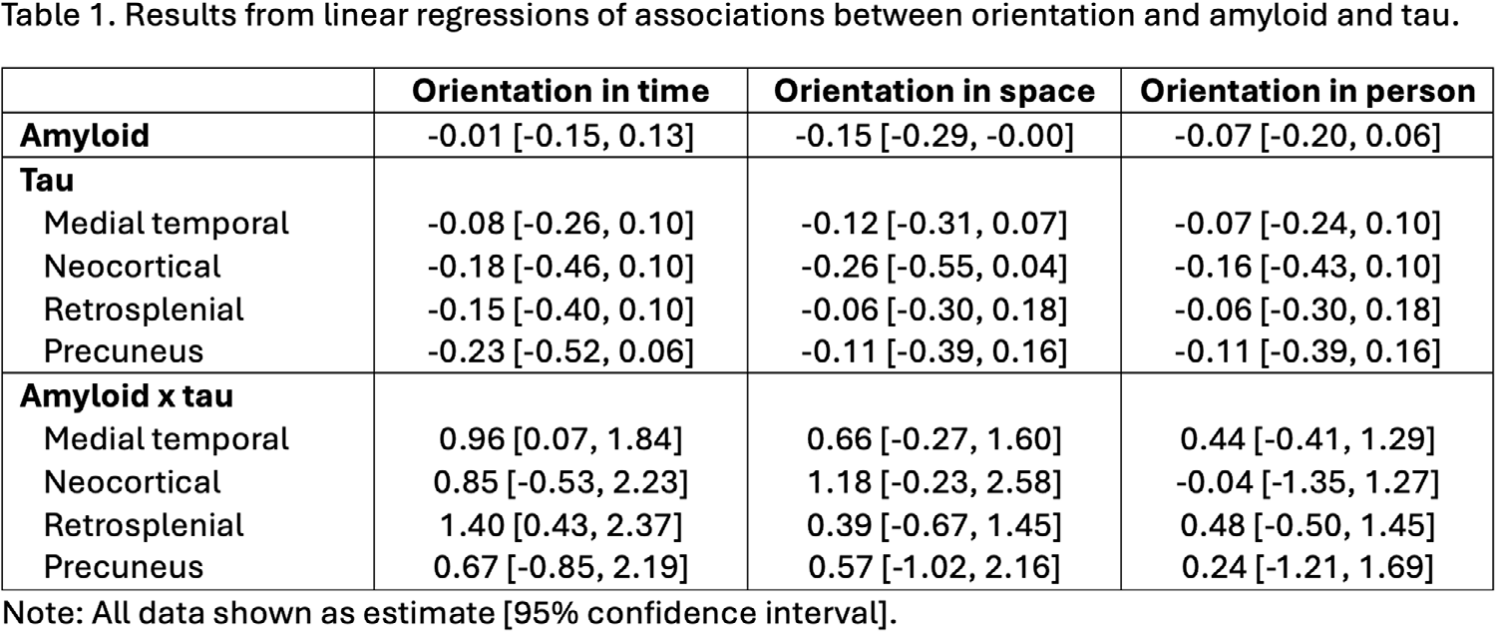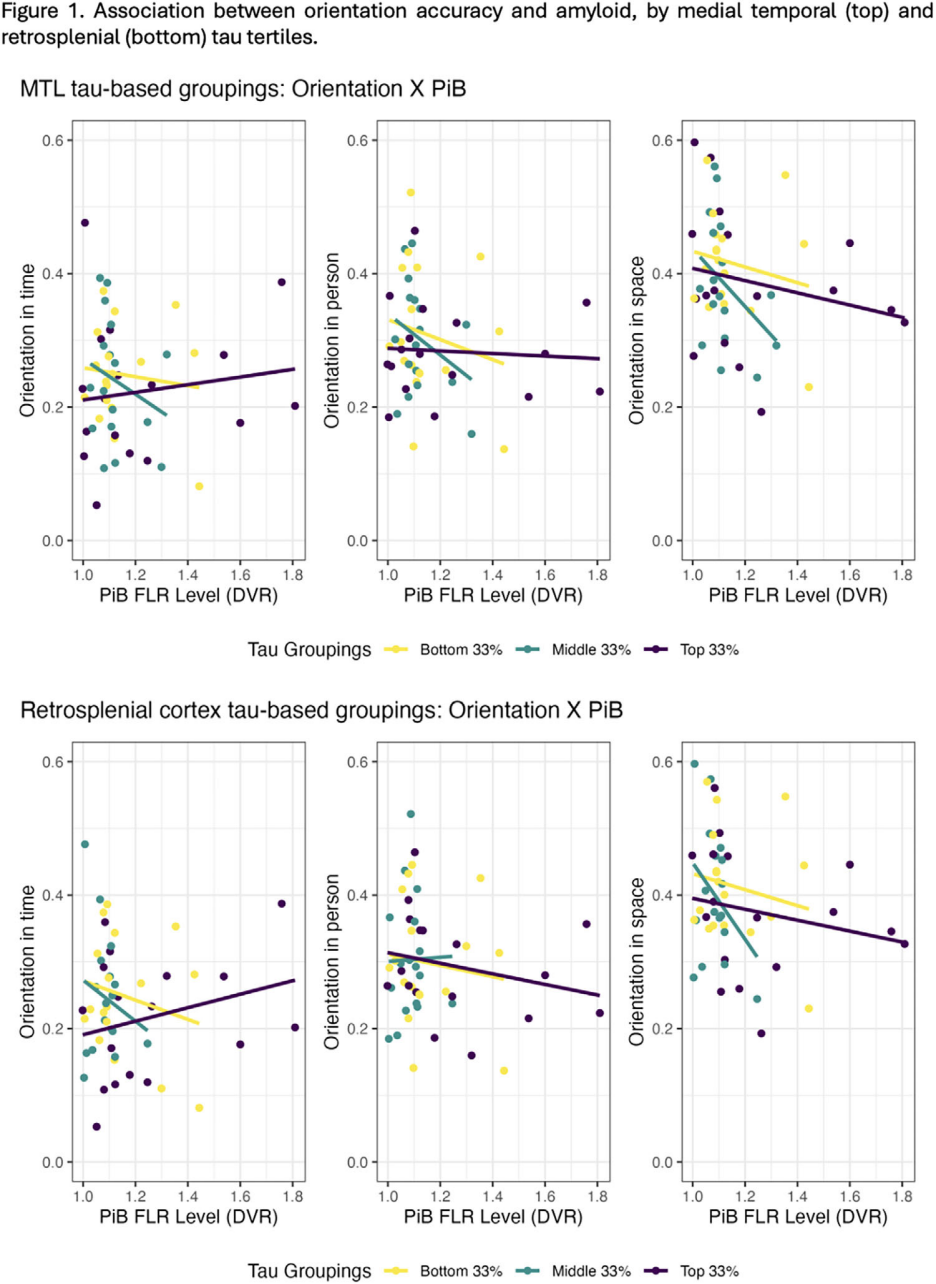# A novel digital paradigm of orientation is associated with amyloid and tau among cognitively unimpaired individuals

**DOI:** 10.1002/alz.084246

**Published:** 2025-01-03

**Authors:** Mark A. Dubbelman, Uri Elias, Phebe Palmer, Amnon Dafni‐Merom, Rebecca E Amariglio, Kathryn V Papp, Onyinye Udeogu, Sharon Wang, Shahar Arzy, Gad A Marshall

**Affiliations:** ^1^ Massachusetts General Hospital, Boston, MA USA; ^2^ Brigham and Women’s Hospital, Boston, MA USA; ^3^ Harvard Medical School, Boston, MA USA; ^4^ Hebrew University of Jerusalem, Jerusalem, Jerusalem Israel; ^5^ Hadassah Hebrew University Medical School, Jerusalem, Jerusalem Israel

## Abstract

**Background:**

Orientation in time, space, and person is often impaired in Alzheimer’s disease dementia. Subtle changes in orientation may occur in prodromal and preclinical disease stages. Here we aim to assess the cross‐sectional association between individuals’ orientation to their own world, as measured with a novel AI‐based paradigm, and Alzheimer’s disease biomarkers (amyloid and tau) in cognitively unimpaired older adults.

**Methods:**

Using an automated chatbot, 51 participants (74.2 ± 5.3 years, 57% female) provided details about personal memories and relationships (orientation in person), as well as recognition of dates of historical events (orientation in time) and geographical locations (orientation in space). Then, they answered ten questions for each of the three domains of orientation. Orientation accuracy was defined as the proportion of correct responses to these questions divided by the time taken to respond to all questions for each domain separately. All participants underwent Pittsburgh compound‐B (amyloid) and flortaucipir (tau) positron emission tomography (PET) scans. We analyzed the relationship between performance on the three orientation domains and the following PET regions of interests: global cortical amyloid and neocortical, medial temporal, retrosplenial, and precuneus tau. All models are adjusted for age, sex, and education.

**Results:**

Higher global amyloid burden was significantly associated with worse orientation in space, while interactions between amyloid and medial temporal and retrosplenial tau were significantly associated with orientation in time (see Table 1). Orientation in person was not associated with amyloid or tau. Figure 1 displays the relationship between orientation accuracy and amyloid for tertiles of medial temporal and retrosplenial tau.

**Conclusion:**

We found associations between orientation in space and time, but not person, and amyloid and tau, even in this small sample of individuals who were cognitively unimpaired, in agreement with previous work, which has shown that orientation in space and time deteriorates earlier in the course of Alzheimer’s disease than orientation in person. These results suggest disorientation to be a core cognitive impairment along the Alzheimer’s disease continuum and may infer a role for routine assessment of orientation, assessed using a personally tailored digital tool, in early diagnosis of Alzheimer’s disease.